# Geographic Association of *Rickettsia felis*-Infected Opossums with Human Murine Typhus, Texas

**DOI:** 10.3201/eid0806.010350

**Published:** 2002-06

**Authors:** Ardys Boostrom, Magda S. Beier, Jackie A. Macaluso, Kevin R. Macaluso, Daniel Sprenger, Jack Hayes, Suzan Radulovic, Abdu F. Azad

**Affiliations:** * Corpus Christi-Nueces County Department of Public Health, Corpus Christi, Texas, USA; † University of Maryland School of Medicine, Baltimore, Maryland, USA; ‡ University of Texas School of Public Health, San Antonio, Texas, USA

**Keywords:** Rickettsia typhi, Rickettsia felis, Murine typhus, cat flea, opossum, serosurvey, PCR, RFLP, Corpus Christi, Texas

## Abstract

Application of molecular diagnostic technology in the past 10 years has resulted in the discovery of several new species of pathogenic rickettsiae, including Rickettsia felis. As more sequence information for rickettsial genes has become available, the data have been used to reclassify rickettsial species and to develop new diagnostic tools for analysis of mixed rickettsial pathogens. R. felis has been associated with opossums and their fleas in Texas and California. Because R. felis can cause human illness, we investigated the distribution dynamics in the murine typhus–endemic areas of these two states. The geographic distribution of R. felis-infected opossum populations in two well-established endemic foci overlaps with that of the reported human cases of murine typhus. Descriptive epidemiologic analysis of 1998 human cases in Corpus Christi, Texas, identified disease patterns consistent with studies done in the 1980s. A close geographic association of seropositive opossums (22% R. felis; 8% R. typhi) with human murine typhus cases was also observed.

Murine typhus is a more common infectious diseases in south Texas. Often the disease is mild and unrecognized; however, it can be severe and even fatal. The severity of murine typhus infection has been associated with old age, delayed diagnosis, hepatic and renal dysfunction, central nervous system abnormalities, and pulmonary compromise. Up to 4% of hospitalized patients die (*1*–*3*). Murine typhus, which is endemic in many coastal areas and ports throughout the world, is one of the most widely distributed arthropodborne infections. Sporadic outbreaks of murine typhus have been reported in Australia and more recently in China, Greece, Israel, Kuwait, and Thailand (*4*–*6*).

Recent serosurveys have demonstrated a high prevalence of antibodies to typhus group Rickettsiae in humans living in Asia and southern Europe. In the United States, thousands of human cases were reported annually in the 1940s (*1*,*2*). A major public health measure consisting of a combination of environmental modification, rat, and vector-control programs greatly reduced human cases in the United States to <100 reported cases of murine typhus/year. As a result, most states no longer report murine typhus. However, murine typhus has been a reportable disease in Texas for the past 40 years.

Interest in this disease has been rekindled because of the resurgence of human cases of murine typhus in south Texas from 1980 through 1984, when 200 cases were reported to the Texas Department of Health. Twenty-eight percent of the patients resided in Nueces County, where the highest annual incidence rate, 4.2 patients/100,000 residents, was reported. Although onset of symptoms occurred throughout the year, 40% of cases were reported in April, May, and June. These studies (Boostrom et al., unpublished; 7–8) also showed that the maintenance and transmission of *Rickettsia typhi*, the etiologic agent of murine typhus, did not occur by the classic cycle involving rats (*Rattus rattus* and *R. norvegicus*) and the rat flea, (*Xensopsylla cheopis*). Detailed investigations of murine typhus in the Nueces County/Corpus Christi area have shown a cardinal role for the opossum (*Didelphis virginiana*) and the cat flea (*Ctenocephalides felis*) in the *R. typhi* life cycle (*7*,*8*). In addition to *R. typhi*, sampled opossums and their fleas were also infected with *R. felis* (formerly known as ELB agent [*9**–**12*]). Furthermore, in 1994 *R. felis* was detected by polymerase chain reaction (PCR) in a blood sample from a patient diagnosed with murine typhus. The presence of *R. felis*, clinically masquerading as dengue fever, was documented recently in patients from Yucatan, Mexico, and four patients with fever and rash in France and Brazil (*13*–*15*). Our published data and these recent reports not only support the pathogenic role of *R. felis* but also demonstrate its wide geographic distribution.

In this study, we report the presence of *R. typhi* and *R. felis* in opossums and their fleas collected during 1998 in south Texas. Data from our 1998 studies show that the rate of seropositive opossums and infected fleas, as well as the *R. typhi*/*R. felis* ratio, are comparable with those in our 1993 studies. In addition, we analyzed the reported cases of murine typhus in Corpus Christi in relation to opossum distribution and seroprevalence. We found a positive correlation between 1998 human murine typhus cases and the geographic distribution of seropositive opossums and their fleas.

## Materials and Methods

### Review of Human Murine Typhus Cases

Historical data on cases of murine typhus are available through the Texas Department of Health and the Corpus Christi-Nueces County Health Department. Extant data fit the confirmed case definition of a fourfold rise in indirect immunofluorescence assay (IFA) titer or a single titer of >1:128 with clinical symptoms. The 1997 data were extracted from cases reported to the Texas Department of Health. In 1998, data included passive and active surveillance of Spohn Hospital System records. In addition, a board-certified infectious disease specialist contacted area physicians about a human typhus study, which was running concurrently with the opossum study. We also included murine typhus cases reported by area physicians during May through July 1998. Data were analyzed for trends in yearly case rate and incidence by age groups. The 1997 and 1998 data were analyzed for sex, age, symptoms, and geographic distribution of cases.

### Opossum Collection

The sera analyzed in this study came from the opossums trapped by Corpus Christi residents during an 18-day period in mid-June 1998. A total of 149 opossums were given to animal control officers for euthanization. Opossums were removed from traps, tagged, and transported to the Vector Control facility, where they were numbered and anesthetized with a ketamine/xylazine mixture. The opossums were weighed, identified by age and sex, processed for ectoparasites, and bled by cardiac puncture. Fleas and ticks were removed with a flea comb. The ectoparasites were collected and placed in vials containing 70% ethanol.

### Rickettsial Seroprevalence in Opossums

Over 95% of trapped opossums were used for a seroprevalence study of rickettsial infections. Initial screening of opossum serum samples of antibodies to *R.* *typhi, R. rickettsii,* *Coxiella burnetii,* and *Ehrlichia chaffeensis* was carried out at the University of Texas at San Antonio. Rickettsial diagnosis was performed with Multi-Test INDX R3E2 Dip-S-Ticks test strips (Integrated Diagnostics, Inc., Baltimore, MD). The assay uses a four-step enzyme-linked immunoassay dot technique for detecting both immunoglobulin (Ig) G and IgM antibodies. Serum samples from uninfected murine typhus patients were used as negative and positive controls. A titer >1:32 was considered positive for *R. typhi*. Eighty samples with equivocal results were retested by the kit manufacturer (Integrated Diagnostics, Inc.). In addition, opossum sera were tested by IFA for antibodies to *R. typhi* and *R. felis* by IFA. Briefly, *R. felis*-infected flea midguts (FleaData, Inc, Freeville, NY) were dissected and placed into individual wells of a 10-well Teflon-coated antigen slide at two midguts/well and allowed to air dry for 20 minutes. Slides were fixed in ice-cold acetone for 10 minutes, air dried, and incubated with individual opossum serum samples (diluted 1:64 and 1:128 in phosphate-buffered saline [PBS]), for 1 hour in a humidified chamber at 37°C. Serum was removed by aspiration, and wells were washed three times with PBS. Midguts were then incubated with secondary antibody (fluorescein isothiocyanate-conjugated goat anti-opossum IgG, [Bethyl Lab., Montgomery, TX], diluted 1:20 in PBS/0.01% Evan's blue) for 30 minutes at room temperature. After three PBS washes, slides were air dried and screened for seropositivity. *R. typhi*-infected Vero cells were also used for the serologic screening. Murine typhus convalescent-phase serum, *R.typhi*-positive opossum serum, negative control serum, and uninfected flea midguts (IFA and PCR negative) were used as positive and negative controls. The cat fleas, purchased from FleaData, Inc., were constitutively infected with *R. felis* (>95% [*15*]) and used as positive controls and antigen sources for opossum serology. The IFA slides were screened by two readers for accuracy. Although attempts to isolate Rickettsiae from the serum samples during the acute phase of infection were unsuccessful, we extracted DNA from selected opossum serum samples. DNA was extracted from 200-µL serum samples by using QIAmp DNA Blood Mini Kit (Qiagen, Valencia, CA) and used for PCR with *Rickettsia*-specific primers.

### Detection and Identification of Rickettsiae in Fleas

Detection and identification of rickettsial species in fleas collected from opossums were carried out using PCR and restriction fragment-length polymorphism analysis of PCR products. Detection of *R. felis* gene encoding 17-kDa protein antigen in fleas was done by PCR as described (*9*–*11*). Briefly, DNA from fleas was obtained by grinding the fleas with grinders containing 20 µL of sterile distilled H_2_O and boiling the lysate for 10 minutes. After centrifugation, 5 µL of the supernatant containing DNA was used for PCR. The DNA template was added to a solution containing 18 µL of PCR Master mix (Roche, Mannheim, Germany) and 1 µL each of forward and reverse primers (100 µmol). In a PCR thermal cycler (Thermo Hybaid, Franklin, MA), each sample was heated to 94°C for 3 minutes, followed by 30 cycles of 94°C for 45 seconds, 55°C for 45 seconds, 72°C for 45 seconds, and an additional incubation period of 72°C for 5 minutes on the final cycle. The target PCR product was visualized by electrophoresis on a 1% agarose gel stained with ethidium bromide and excised; DNA was recovered from the gel with a StrataPrep DNA extraction kit (Stratagene, La Jolla, CA) according to manufacturer's protocol. Enzymatic digestion of cleaned PCR product was done by incubating 8 µL of DNA in 1X enzyme buffer (10 mM Tris-HCl [pH 7.5], 50 mM KCl, 0.1 mM EDTA, 1 mM dithiothreitol, 200 µg/mL bovine serum albanin, and 50% glycerol), and 15 U of *Alu*I (Stratagene) for 1 hour at 37°C. Digested products were visualized on 8% TBE gels (Novex, San Diego, CA) stained with ethidium bromide. For sequencing, the purified 17-kDa fragments were subcloned in TOPO TA cloning vector (Invitrogen, San Diego, CA) and were sequenced by the dye terminator method on a model 373 automated fluorescence sequencing system (Applied Biosystems, Foster City, CA). Sequence analysis was performed with the MacVector software package (Accelrys, Inc., Madison, WI), and the BLAST program (National Center for Biotechnology Information, Bethesda, MD) was used for comparison. Sequencing was carried out three times, in both directions, to ensure fidelity.

## Results

### Human Murine Typhus Cases, Corpus Christi, Texas

Since the 1970s, the number of murine typhus cases has fluctuated around 20 cases/year in south Texas. In 1997, however, a record number of cases, 72, were reported in Texas, resulting in a statewide incidence of 0.4/100,000 population. Sixty-nine of the 72 cases occurred in Region 11 of the Texas Department of Health; most cases occurred in three counties, Hidalgo, Cameron, and Nueces. These three counties consistently register the majority of murine typhus cases in Texas. Data from January 1985 through December 1997 show that Nueces County has averaged the most cases. Cases are reported year-round; however, peak incidence occurs during May and June, which leads local physicians to call murine typhus “the summer flu.” Murine typhus cases from 1997 and 1998 (Figure 1), which occurred in residents of Corpus Christi, were reviewed. Patients ranged from 5 to 79 years of age (mean 40 years). The 1997 and 1998 murine typhus patients were analyzed for race, ethnicity, history of fleabite, exposure to cats and opossums, and presence of symptoms. Fifty-five percent of patients were Hispanic, and 62.2% were female. Symptoms included headache (56%), fever (100%), rash (27%), nausea/vomiting (51%), malaise/fatigue (44%), arthralgia/myalgia (22%), and diarrhea (20%). Fewer than 15% of patients reported a history of fleabite, and the exposure to cats or opossums at residences was associated with only 13% and 11% of cases, respectively. Nueces County/Corpus Christi had 14 of the 42 confirmed murine typhus cases reported in 1999 in Texas and 20 of the 52 reported cases in 2000.

### Characteristics of Opossums Trapped for Typhus Studies

Opossums are nuisances for residents of Corpus Christi by inhabiting den sites in junk heaps, storage sheds, garages, and attics. Corpus Christi’s opossum population is controlled primarily by private citizens using personal traps. Fifty traps are available at nominal rental through the Corpus Christi Animal Control Program. In contrast, anecdotal information from the nearby Flour Bluff and Calallen areas suggests that residents in these areas tolerate opossum presence. Most opossums that cause problems for residents in these areas are destroyed privately; occasionally, they are used for food. Nevertheless, from 1996 through 1998, Corpus Christi Animal Control trapped and euthanized >18,000 opossums. The mean number of trapped opossums during this 3-year period was 6,324/year. Although data regarding opossum population size, based on the average number of trapped opossums/year, are not available for the study area, the trapped population may represent 20% to 30% of the total yearly population. If this is the case and assuming equal distribution of opossums’ ideal habitats throughout the city, the opossum population density in Corpus Christi could approach >75 opossums/square mile or approximately 1 opossum/0.013 square mile.

Although opossums are collected continuously in the Corpus Christi area, the 1998 study focused on opossums trapped within approximately a 3-week period during the traditional peak of human murine typhus cases. The characteristics of the 149 opossums trapped during June 8–25, 1998, were as follows: 51% female (n=76); 49.0% (n=73) male; and 47.7% juveniles and sexually immature (Table 1). Weight of the trapped opossums ranged from 5 oz to 8 lbs (mean weight 13 oz for juveniles; 4 lbs 14 oz for adults).

**Table 1:** Rickettsial seroprevalence in 149 opossums, Corpus Christi, Texas

	Female	Male
Age	Positive/total (%)^a^	Positive/total (%)^a^
Juvenile	7/31 (23)	10/40 (25)
Adult	15/45 (33)	6/33 (18)
Total	22/76 (29)	16/73 (22)

^a ^Enzyme-linked immunoassay (Integrated Diagnostics, Inc., Baltimore, MD)

### Rickettsial Seroprevalence in Opossums

In 1998, a seroprevalence study for *R. typhi* showed a geographic association between human cases of murine typhus and ranges of seropositive opossums (Figure 2 and Figure 3). Six (31.6%) of the 19 patients lived within the minimum home range, 0.02 square mile, of a seropositive opossum. Another five patients (26.3%) were within the maximum home range, 0.1 square mile, of a seropositive opossum. Initial studies on seroprevalence of rickettsial infections in opossums carried out by enzyme-linked immunoassay showed no seroreactivity to *C. burnetti*, the agent of Q fever; *E. chaffeensis*, the agent of monocytic ehrlichiosis; and *R. rickettsii*, the agent of Rocky Mountain spotted fever. However, >25% of the 149 serum samples reacted with *R. typhi* antigens (Table 2). Seventeen (23.9%) of juvenile and 21 (26.9%) of adult opossums were seropositive (Table 1). Reevaluation of the opossum serum samples by using IFA with both *R. typhi* (Wilmington strain) antigens and the *R. felis*-infected cat flea midguts, showed that 8% and 22% of opossum sera were reactive at >1:128 with *R. typhi* and *R. felis,* respectively (Table 2). Although *R. felis*-infected flea midguts were used to identify non-*R. typhi* seropositive opossums, these two rickettsial species could not be distinguished in some samples (n=6). Since both *R.typhi*- and *R. felis*-positive fleas were collected from opossums, the possibility of dual infections of opossum could not be ruled out, even though dual rickettsial infection in fleas has not been reported (*16*).

**Table 2:** Seroprevalence of *Rickettsia typhi* and *R. felis* in 149 opossums collected in Corpus Christi, Texas, June 1998^a^

Opossum serum samples	Serologic assays	
	Positive/total (%)	
	*R. typhi*	*R. felis*
EIA	38/149 (25)	N.D.
IFA^c^	10/125 (8)	28/125 (22)

^a^ EIA, enzyme-linked immunoassay (Integrated Diagnostics, Inc., Baltimore, MD); N.D., not done; IFA, indirect immunofluorescence antibody assay. ^c^ IFA with *Ctenocephalides felis *(FleaData *R. felis-*infected colony) midgut smears and *R. typhi* (Wilmington strain) as antigens at >1:128 titer.

### Detection and Identification of Rickettsiae in Cat Fleas

A total of 3,401 fleas were collected from 147 opossums. Over 99% of the fleas collected were identified as *Ctenocephalides felis*. The number of fleas per opossum ranged from 1 to 488 (mean 23 fleas/opossum). Initially, ricksttsial infection in fleas was assessed by using IFA with rat polyclonal anti-*R. typhi.* A total of 359 fleas collected from 50 opossums were sampled and tested individually by IFA; 20% of the fleas were positive. PCR was used to confirm rickettsial infection in fleas. Analysis of 529 individual fleas from 144 opossums showed an overall infection rate of 2.6% (14 confirmed positive). Restriction fragment-length polymorphism analysis of positive flea PCR products yielded a banding pattern representing 3 *R. typhi*- and 11 *R.felis*-infected fleas (Figure 4). The overall *R. felis* infection rates for 1998 samples were lower than 1993 infection rates (Table 3). Overall, 8% of the opossums had positive fleas when fleas were tested individually, compared with 21% when flea pools (50 pools; <20 fleas/pool/opossum) were used. The observed discrepancy between the results from pooled and individual flea samples reflects the variability in the DNA recoverable by PCR procedure. Although there was a positive correlation between the opossum age and the flea/opossum ratio, infected fleas came from both juvenile and adult opossums. Additionally, no correlation between the infected fleas and seropositive opossums existed.

**Table 3:** *Rickettsia typhi* and *R. felis* infections in opossums and their fleas, Corpus Christi, Texas

	Total	*R. typhi*	*R. felis *
Sample	Positive/total (%)	Positive/total (%)	Positive/total (%)
Opossum			
1993^a^	3/9 (33)	0/3 (0)	3/9 (33)
Cat flea^a^			
1993	18/399 (5)	3/399 (1)	15/399 (4)
1998	14/529 (3)	3/529 (1)	11/529 (2)

^a^Confirmed with polymerase chain reaction/restriction fragment-length polymorphism sequencing.

## Discussion

Since 1946, the Annual Summary of Notifiable Diseases in Texas has included murine typhus. Historical data identifies 1,127 cases of murine typhus in 1946. However, the reported cases of murine typhus dropped rapidly with the advent of successful rodent and flea controls; by 1952, <100 cases/year in Texas were reported (*1*,*2*). Through 1960, the number of human cases steadily decreased, ranging from 12 to 50 and averaging 20 cases/year. The sudden increase in locally acquired cases in the 1990s presented a different reservoir-vector-rickettsia paradigm. Historically, murine typhus infection as an urban zoonosis has been maintained and transmitted in commensal rodents, in particular the Norway rat (*R. norvegicus*), and the oriental rat flea (*X. cheopis*) (*1*,*2*). However, in recent years the zoonotic cycle responsible for the documented human murine typhus cases in south Texas, as well as southern California, has been shown to involve opossums and cat fleas (*7*–*10*,*17*). The role of opossums and cat fleas in the transmission of *R. typhi* in suburban focus of murine typhus in Los Angeles County has been well documented (*9*,*17*). As in our study, a high proportion of opossums collected in Orange County, California, was seropositive for rickettsia (*9*,*17*). Opossums, as a peridomestic animal, are frequent visitors of human habitations, where they search for both harborage and food and thus expose the occupants to cat fleas and consequently to rickettsial pathogens. Cat fleas are frequently found in large numbers on opossums and are avid feeders on humans and household pets. In addition to *R. typhi,* the cat fleas also harbor *R. felis*. In fact, the cat flea infection with *R. felis* is more common than *R. typhi* (*7*,*8*,*10*). Both rickettsial species are readily maintained transovarially in fleas (*11*,*12*,*18*), but in contrast to commercial cat flea colonies that usually maintain >80% *R. felis*-infection rates, only 1% to 5% of wild-caught fleas are infected with this rickettsial species (*7*,*8*,*10*,*18*).

We have shown that cat fleas collected from opossums from Corpus Christi, Texas, were infected with either *R. typhi* or *R. felis,* and the infection rates remained <5% in both the 1993 and 1998 samplings. While we have found no evidence for dual infection in individual fleas, opossums fed on by infected fleas could have antibodies against both *R. typhi* and *R. felis*. Our initial opossum serosurvey results (Table 1 and Table 2) using enzyme-linked immunoassay were directed against *R. typhi* only. However, IFA results from *R. felis*-infected fleas confirmed our earlier findings (*7*,*8*,*10*) that the cat flea/opossum cycle is responsible for the maintenance of both *R. typhi* and *R. felis* in Corpus Christi.

We have reported the importance of *R. felis* as a component of murine typhus transmission cycles (*14*,*19*,*20*). Both *R. typhi* and *R. felis* were found in fleas and opossum tissues from the murine typhus–endemic areas of southern California and south Texas (*7*,*8*,*10*). Additionally, a retrospective investigation of five murine typhus patients from Texas demonstrated that four of the patients were infected with *R. typhi* and the fifth had been infected with *R. felis* (*7*). This documented human infection with *R. felis* and its presence in opossums and their fleas, and possibly in other wildlife associated with human habitations, have raised concerns about *R. felis* spillover into human populations. In addition, cat fleas infected with *R. felis* have been identified not only in the United States (*19*) but also in Central and South America, Europe, and Australia (T. Kilminster, unpub. data).

Together, our published data and these recent reports not only support the pathogenic role of *R. felis* but also demonstrate its wide geographic distribution. However, we know very little regarding the natural maintenance and transmission of this organism in areas of the world besides south Texas and southern California. The cat flea, known as an indiscriminate feeder, has an extremely broad host range. While it parasitizes cats, opossums, and other animals of the same size, the flea readily switches to different hosts, and it has been found on rats and mice. Because cat fleas are commonly found on household pets, we extended our studies to determine rickettsial seroprevalence in cats. Our pilot serologic studies showed >15% of 513 serum samples from the eastern USA were reactive at >1/64 with *R. felis*, as assessed by IFA (Higgins et al., unpub. data). Sorvillo et al. (*17*), in their Los Angeles study of a suburban focus of murine typhus, reported that 9 of 10 domesticated and 3 of 26 feral cats were seropositive to *R. typhi*. Thus, domesticated cats and cat fleas, as well as peridomestic animals, may play an important role in the maintenance cycle of *R. felis* and its transmission to humans. Our study further documents the involvement of the opossum/Rickettsia/cat flea triad in the flea-associated rickettsial transmission cycle of urban and suburban areas of south Texas and southern California. Similar host/parasite relationships may also operate in other parts of the world where recent *R. felis* human cases have been documented (*13*,*15*). Recent attention to *R. felis*, which already has resulted in reassignment of this organism to the spotted fever group rickettsiae (*14*,*15*), may further elucidate the other components involved in the maintenance of this rickettsiosis.
